# The complexities of ‘otherness’: reflections on embodiment of a young White British woman engaged in cross-generation research involving older people in Indonesia

**DOI:** 10.1017/S0144686X14001366

**Published:** 2014-12-18

**Authors:** MERIEL NORRIS

**Affiliations:** * Centre for Research in Rehabilitation and Brunel Institute of Healthy Ageing, Brunel University, London, UK.

**Keywords:** embodiment, intersectionality, intersubjectivity, ethnicity

## Abstract

If interviews are to be considered embodied experiences, than the potential influence of the embodied researcher must be explored. A focus on specific attributes such as age or ethnicity belies the complex and negotiated space that both researcher and participant inhabit simultaneously. Drawing on empirical research with stroke survivors in an ethnically mixed area of Indonesia, this paper highlights the importance of considering embodiment as a specific methodological concern. Three specific interactions are described and analysed, illustrating the active nature of the embodied researcher in narrative production and development. The intersectionality of embodied features is evident, alongside their fluctuating influence in time and place. These interactions draw attention to the need to consider the researcher within the interview process and the subsequent analysis and presentation of narrative findings. The paper concludes with a reinforcement of the importance of ongoing and meaningful reflexivity in research, a need to consider the researcher as the other participant, and specifically a call to engage with and present the dynamic nature of embodiment.

## Introduction

Gerontologists have long been concerned with social identities, but specialism has encouraged a focus on predominantly one dimension of social identity, in this case age (Torres 2015). While increasingly challenged through concepts of intersectionality (Koehn *et al.* 2013) and demonstrated, for example, through the emerging focus on ethnicity within gerontological research, this multi-dimensional view has been primarily applied to understanding the narratives of participants in research. Within qualitative literature, limited consideration has been given to intersectionality and the social identity of the researcher together with the potential influence that may have on the research process (Ellingson 2012; Turner and Norwood 2013). The purpose of this paper is to present a theoretical background for why a focus on the researcher should be a critical methodological concern in gerontology which engages with ethnicity, specifically illustrating its relevance through examples in a cross-ethnic and cross-generational context.

Social scientists, including gerontologists, have deconstructed categories such as age, gender and ethnicity, illustrating not only how they are constructed socially but also how they overlap, change and are negotiated (Brunsma, Delgado and Rockquemore 2013; Butler 1993; Hawson 2013; Kannen 2013; Phillipson 2008; Torres 2009; Twigg 2006). Hence researchers have illustrated how the experience of ageing can be mediated by minority ethnic status, but within that, specific influences associated with gender (Bauer and Thompson 2006; Gardner 2002; Saltus and Pithara 2014; Victor, Martin and Zubair 2012; Wray 2003, 2004) and migration experience (Cook 2010; Phillipson, Ahmed and Latimer 2003; Saltus and Pithara 2014; Torres 2006), amongst many others. This deepening insight into the complexity of social identities and practices has highlighted a concern with intersectionality in the consideration of research participants. While the term in the North American context has been associated with combinations of social disadvantage (Koehn *et al.* 2013), within the United Kingdom (UK) it has come to represent a broader appreciation that people cannot be reduced to single descriptive categories (Phoenix and Pattynama 2006). In doing so, researchers have illustrated the complexity of older peoples' lives informed and influenced by the multiple social identities that are formed and reformed throughout their lives.

While Torres (2015) notes that this approach remains limited in gerontological research on ethnicity, to date this richer conceptualisation of the participant has also rarely been afforded to the interviewer. As such, descriptions of the researcher themselves, if given at all, are generally limited to simple categorisations (*e.g.* Moffatt and Mackintosh 2009, in which the interpretation process is described in detail but the researchers themselves are not; and Doshani *et al.* 2007 who describe the researcher as linguistically matched, with no further information). Authors suggest that such minimal information potentially results in interviewer anonymity and misplaced assumptions of neutrality (Antelius 2009; Brown and Boardman 2011; Dunbar, Rodriguez and Park 2002; Sharma, Reimer-Kirkham and Cochrane 2009). Indeed, in the field of cross-ethnic research, authors suggest that the ascribed and interpretable ethnicity of the researcher becomes hidden behind the focus on the participant (Ellingson 2006; Torres 2009; R. Willis 2012). Concern with this limited exploration and presentation of the researcher in gerontological research is highlighted when the importance of the interaction between participant and researcher is considered.

Qualitative researchers have increasingly engaged with the complex interactions between participants and the researcher. For some this has resulted in consideration of particular social identities or body signifiers ascribed to the participant or researcher such as gender, age, ethnicity, class and disability in relative isolation (Brown and Boardman 2011; Manderson, Bennett and Andajani-Sutjahjo 2006; Seymour 2007). Such an approach often presents researchers and their participants either in terms of their difference – otherness – or similarities, and outlines the perceived consequence in terms of access, data production or interpretation. Methodologically, apparent differences in these defined categories have been problematised as creating potential social boundaries which are perceived to influence negatively the researcher–participant interaction and the subsequent data collected (Manderson, Bennett and Andajani-Sutjahjo 2006). Approaches such as gender, age and more recently ethnic matching have been proposed to address such concerns (*e.g.* Bhopal 2001; Doshani *et al.* 2007; McLean and Campbell 2003; Moffatt and Mackintosh 2009; Shanley *et al.* 2013). However, the ability of matching to enhance the research encounter has come under scrutiny.

While acknowledging the importance of such studies and the potential utility of matching, critics argue that the focus on single attributes potentially masks the interplay of different identities and the shifting nature of these identities within emerging and developing relationships (Sharma, Reimer-Kirkham and Cochrane 2009; Turner and Norwood 2013). Indeed, in the field of ageing and ethnicity, studies have illustrated the complexity of ethnic and gender matching and how assumptions of ‘sameness’ or ‘difference’ between researcher and participant cannot be accounted for within a single category (Wray and Batholomew 2010; Zubair, Martin and Victor 2012*a*, 2012*b*). Focus is drawn to the collaborative, intersubjective nature of the interview process and outcome and intersectionality of both participant and researcher (Ben-Ari and Enoush 2013; Ellis and Berger 2003; Jones 2006).

Debates continue regarding how the intersubjective encounter between researcher and participant can be enhanced within interviews. Finlay (2005), for example, discusses the need for empathy with an intertwining of the worlds of participant and researcher. Zahavi (2005) highlights the existence of a common world when empathy is not generally required as shared experience, actions and traditions facilitate engagement. In apparent contrast, he also acknowledges that intersubjectivity may necessitate ‘confrontation with radical otherness’ (Zahavi 2005: 168) through which the awareness of the objectivity of self facilitates experience of the other. Each approach highlights a need to engage and explore the self (researcher) and authors have highlighted the need to move beyond language in such an endeavour to consider the essential role of embodiment (Broom, Hand and Towey 2009; Finlay 2005, 2006; Sharma, Reimer-Kirkham and Cochrane 2009).

The concept of embodiment encourages a focus on the body as the site of perception of self and other (Finlay 2005, 2006; Sharma, Reimer-Kirkham and Cochrane 2009; Zahavi 2005) and there has been an increasing area of interest in the concept within gerontological research (*e.g.* Clarke and Bennett 2013; Clarke and Griffin 2008; Twigg and Buse 2013). Consequently, who and what I am at a specific time and place is lived through and performed by my body, which is itself culturally and historically influenced (Brown *et al.* 2011). In addition to the language shared within interviews, authors argue that embodiment has the potential to influence the research encounter and therefore its presence must be made explicit (Kannen 2013). By taking note of embodiment, the internal and external perception of the researcher's being in the world can be explored and, through this, that the world of the participant can be placed within a situational context (Sharma, Reimer-Kirkham and Cochrane 2009).

Reflexivity within research has been posited as an appropriate method to interrogate embodiment in order to illuminate its influence on the production and presentation of stories and meanings (Alvesson and Skoldberg 2009; Finlay 2006; Sharma, Reimer-Kirkham and Cochrane 2009). In this context, I draw on both Schwandt's (2007) description which emphasises not only critical self-reflection but significantly how that awareness informs the entire research process, and Doyle's (2013) assertion that reflexivity is intersubjective in that it involves both the researcher and participant and requires a capacity to utilise the insights gained. However, how the embodied researcher influences the research encounter and the resultant data and analysis remains relatively under-explored or under-reported (Turner and Norwood 2013; Underwood, Satterthwait and Bartlett 2010) and Clarke (2012) has called for increasing consideration of the embodied researcher in gerontology.

In the field of ageing and ethnicity such engagement has been very limited, but studies have served to highlight the complexity and fluctuating nature of embodiment and its influence on the research process and resultant data produced (Higgins 1998; Zubair, Martin and Victor 2012*a*, 2012*b*). These studies detail the need to consider not just ethnicity or age or gender, for example, but how these characteristics are interpreted, modified and interplay with both the physical and social context of the individuals involved at specific moments in time. Such observations are in line with methodological reports which highlight how researchers are conscious of their self-image and multiple identities, can and do adapt their bodily performance during research encounters, and the influence of this on the data that emerge (Giardina and Newman 2011; Hyden and Brockmeier 2008; Lillrank 2012).

To further the debate on the methodological significance of embodiment with specific relevance to ageing and ethnicity, this paper draws on a reflexive embodied account from fieldwork in which the author was both crudely an ethnic and generational ‘other’. The overall purpose is to illustrate the complex and evolving nature of researcher embodiment and the potential methodological significance of its consideration. Through this, apparent differences in age and ethnicity between interviewer and interviewee are shown to be contextually situated in time and place, dynamic, with fluctuating relevance in the process of developing participants' stories. For gerontologists, particularly those with an interest in ethnicity, the examples serve to highlight the need to be aware and make transparent the complexity of themselves in the stories of participants' lives they wish to present.

## Researching in foreign lands: an outline of the study and its context

While most gerontological work is conducted within the researcher's ‘home’ country, rarely does it occur in the researcher's ‘home’ space. Other people's residences, hospitals, care homes, social centres, for example, can be construed as ‘foreign’ spaces for researchers. Considering research in an apparently very ‘foreign’ environment, as was the case in the illustrative research to follow, serves to highlight features of apparent dislocation in place which has relevance but may infrequently be considered in gerontological research more widely.

The study from which this paper is based explored life after stroke in a rural multi-ethnic area of Aceh, Indonesia, drawing on narratives of life and its reconstitution after a critical change in circumstance. Participants included seven women and four men, all stroke survivors, and their carers. All were from lower socio-economic backgrounds. The study involved Gayonese, Javanese and Acehnese participants with related complexities created through histories of forced and optional migration, political alignments and the relatively recent cessation of a 30-year civil war which was predominantly fought on ethnic grounds (for further details, *see* Norris 2009). Many of the participants were older, with seven of the 11 over 50 years (mean age 52 years, range 32–69 years). Life expectancy at the time was under 70 years, ten years below that of the UK (World Bank 2014). The older age of participants is expected as the prevalence of stroke increases with age, but research has also indicated that stroke survivors perceive that they have been catapulted into an older age, highlighting the relevance of gerontology to the topic (Kilbride, Allison and Evans 2011). Aceh is a predominantly Muslim region and *Syariah* law was implemented in 2005.

The fieldwork was conducted over a total of ten months by a 35-year-old unmarried, white British woman. Further relevant details of the researcher's identities are explored later. The research was influenced by both ethnographic and phenomenology traditions, in which the experience of living with stroke within the context of an ethnically diverse and post-conflict community was a primary focus. Methods included participant observation, both general to community and specific to participants, in-depth interviews and repeat interviews with photo-elicitation. Focus groups and vignettes were also used at various stages (for further details, *see* Norris 2009). The researcher was competent in the national language, Bahasa Indonesian, but also worked with a co-researcher who was local and trilingual, as required in this complex research area.

Issues relating to the use of the co-researcher are not addressed in this paper although it is accepted that the addition of another researcher adds further layers of interactive complexity and cross-language research poses significant dilemmas between what is said and what is heard (Ballantyne, Yang and Boon 2013). The complex data were managed through atlas.ti software and the narrative texts were thematically analysed alongside the contextualising field notes (Braun and Clarke 2006). A reflexive diary was kept through the fieldwork, analysis and writing periods which was viewed by the primary researcher alone. Alongside this, regular discussions were held with the supervisory team (two academics not working in the field area) and a local field site team to consider issues highlighted in the diary and aspects of interpretation throughout the research. This paper primarily draws on this diary and discussions, observation notes and textual data from the interviews.

During time in the field and through the analysis phase, who and what I was perceived to be, by myself and the participants, became increasingly important areas of reflection in order to place the current life experiences of older stroke survivors in context. It was apparent that my identity was being constructed in the moment and exploring the nature and critically the influence of this was highlighted as an important methodological concern. In the following section, I focus on three aspects of the embodied researcher within cross-generational and cross-ethnic research: ‘embodied roles’ describes the salience of the student daughter roles in reconstructing the apparent differences in age and social context; ‘embodying emotion’ explores the complexity of situated gender; and ‘embodiment’ focuses on the physicality of the body. While other examples could be drawn on reflecting multiple identities, the three presented specifically challenge concepts of age and ethnicity and other identities that intersect with them, influencing the intersubjective encounter.

## Embodied roles

The first aspect is the intersectionality between roles within the researcher that may play out and influence the research process. All researchers embody multiple roles; daughters, sons, parents, professional, yet these are rarely acknowledged. Exploration highlights how place and context, time, gender and topic amongst others have the potential to support the emergence or suppression of specific roles, but also the dynamic and fluctuating nature between the different embodied attributes. The example below draws specific attention to the interaction between age and roles and the understated influence of ethnicity within the encounter.

Like many gerontologists, at the start of this study I perceived that my primary role was to be a researcher, more specifically a research student. I made efforts to maximise my success in this role and in doing so embodied being a research student. However, awareness of my embodied self during interviews exposed other living identities, as a health professional (in the UK), as a sister, or in the example below a daughter. These intersecting components of my embodied self were not silent in interviews or analysis. They interjected in expected but often unexpected ways and times and in doing so informed the results of the study.

### A conversation about fatherhood: being a student and daughter

The incident described was held in the public room of Pramana's home, a room Pramana had previously used for running informal educational sessions for his and the neighbouring children. As tradition in this region, we all sat on the floor and exchanged general conversation over coffee before the interview began. Pramana, who was a 66-year-old man, was joined by his wife and variably by two of their six children (both daughters) who moved in and out of this communal space. I was dressed appropriately with arms and legs covered, but my hair uncovered as accepted and indeed expected as a non-Muslim in this specific region and household. My notebook and pen were in my hands and the dictaphone lay on the floor in front. I was set physically, practically and intellectually, poised to hear the story Pramana would choose to tell.

While discussing the impact the stroke had on his life, Pramana raised the topic of education. He explained how education was one of the key responsibilities that a parent must provide for their children, but also emphasised that this role was as important to deliver to his daughters as his sons, acknowledging the presence of his daughters as he spoke. This egalitarian view of education has been previously noted in the region (Bowen 1991) and so I was not unduly surprised by this inclusive approach. However, as mentioned above, Pramana was Javanese and I consequently wondered if inclusivity was a previously held commitment or one adapted to the specific environment in which Pramana now lived. This conversational turn to ungendered education alongside the performance of the father–daughter connection was not, however, only an intellectual observation, but also instigated a shift in my experience of myself within the exchange. I became aware that I had until this point positioned myself, both within the conversation but also literally on the floor with my trappings of note taking, as a student, eagerly listening to the teacher who would educate me on their experience, taking notes and asking pertinent questions. I not only positioned myself as a student, I was one, a point not lost on either myself or Pramana who was aware that it was this identity that had brought me to his home. Moreover, while I was somewhat older than most of his students, I was nevertheless over 20 years his junior and in life terms, as unmarried and without children, shared more with his charges than he himself, a situation frequently encountered within gerontological research.

My embodied performance of student as highlighted by my notebook and dictaphone, but also my gentle nodding, attentive eye contact, probing questions, reinforced the value of the topic in question, but also his position as the educator, implicitly encouraging the conversation through our mutually collaborative roles. So while an ‘other’ in age terms, it was an ‘otherness’ that was within a mutually recognisable frame. The shared experience of the teacher–student relationship, albeit with different and complementary roles to play, created a salient mutually recognised platform. This apparently reduced the appearance of ethnic or religious differences as important features. Such gendered and generational roles have been discussed elsewhere in relation to cross-cultural and age scenarios (Rubin 2012). Less has been said about how being a daughter influences the interaction, yet a subtle shift in the conversation that continued with Pramana brought this aspect of my embodied presence to the fore.

As the conversation progressed to confront the loss of this educational role as a consequence of his stroke and the fear that his younger daughters may not be educated as he was now too tired and unwell to provide, this student body I had inhabited was hijacked by a more personal and emotional engagement as a daughter. My thoughts as I listened to this man share his vulnerabilities and fears became peppered by my late father, an academic who had died many years before my education was complete. These thoughts were echoed in the conversation as Pramana shared his hopes that his children could complete their education as I had, and verbalised the pride I imagined my father would have had on completion of my studies. While emotionally disarmed by the unexpected inclusion of my father, I quashed my instinct to verbally react to this statement, allowing the presumed shared connection between Pramana and my deceased father to persist. My engagement with the conversation however altered, I physically softened, rested my hands in my lap and my note taking basically ceased.

Later, reflecting on the conversation, my father reappeared, prompting the following entry in my diary:

All this thinking leads me back to Dad. He crops up so much in my head … I wonder what he would make of all of this – what I'm doing, where I'm doing it and how. Would he have been proud? Guess I'll never know.

During the provisional analysis that followed, the emotional weight of the loss of my father in his educational role and the series of questions I personally still had unanswered heightened. I began to feel the frustration that this imbalance in capacity and social duty created, but also the potential complications for the younger children. Would they resent their older siblings if they were not afforded the same opportunities? How would they relate to their father who until now had provided effectively under difficult social conditions? My emotional connection as a daughter who had experienced this loss, albeit under very different circumstances and place, stimulated a deeper consideration of the consequence of loss, but also how those potentially changing relationships would impact on Pramana. I wondered if he engaged in this forward thinking and if the sadness he expressed was not just for the loss of role as teacher and provider, but also his potential fear of a loss of love, of respect and the instability in the family structure that may evolve. These were topics we had touched on in relation to his need to be cared for, but it was only later that the connections between this cared-for role and the multi-layering of the educational provider role began to merge, stimulated by the merging apparent in my own past experiences. Through this process, the parallels and differences between the two fathers evolved. I further considered the shared roles they held and the desire to complete socially accepted responsibilities, and within this how my ethnic ‘otherness’ was diminished. The differences in vulnerability created by place and socio-economic and political context insured the ‘otherness’ was all too apparent. My education was completed despite my loss of father, whereas for Pramana and his daughters such security was not available.

Within the space of one conversation my embodied presence and experience as a young person, a student, a woman, a daughter bereaved of father and a person socio-economically privileged emerged, dissolved, merged and shifted in unique and temporally situated ways, illustrating the fluctuating and interactive nature of embodiment. Crucially, not only did these shifts potentially result in different stories being shared, but awareness within myself facilitated a more engaged analysis where acknowledgement of our common worlds, but also more radical differences, facilitated a deeper and more emotionally engaged reflection on the words spoken. A focus on any one of these embodied presences alone and/or an assumption that they are static within the evolving relationship between interviewer and interviewee would risk simplifying this dynamic interaction and in doing so the insights gleaned from a deeper engagement.

## Embodying emotion

The second aspect explores more closely the complex area of researcher emotion within the research interaction, an area frequently neglected in research literature (Fitzpatrick and Longley 2014; Willis 2012). Within the following example, I illustrate the intersectionality of my embodied gender, marital status, ethnicity and age alongside that of the participants and how these in combination created fertile but also emotionally challenging ground within the research process. This intersectionality raises questions not only regarding who I wished to be in the context in which I was working, but also what constitutes a story and how the decision of representation is made.

### Gendered vulnerabilities in ageing: women from different worlds

Through the course of the research I met a number of women who had been left by their husbands, or were fearful that they would be deserted and who in some way related these events to their stroke. In contrast, none of the male stroke survivors had been left by their wives. The stories which developed over time were a rare insight into the social dynamics of marriage and how they interplayed with Islamic law and local Gayonese culture. On the one hand, participants explained how land could be owned and worked by women and therefore the potential for security remained if their husband had left them. Yet at the same time, the partner who owned the land was morally responsible to support the other members of the family. Consequently, female stroke survivors whose husbands had left them explained how they had to farm their land not only for their survival and that of their children, but also to provide for their previous husband and in some cases, his new wife. They discussed how this was not official law in the area, but the norms of behaviour between couples were influenced heavily by local interpretations of Islamic doctrine. As women they felt disempowered to question these norms which were in turn reinforced by a strong social pressure to behave socially as a ‘good Muslim’, prohibiting them from open discussion of discontentment of their position (for further discussion on this concept, *see* Norris 2009). These narratives of engendered vulnerability in later life demonstrated a complex mix of cultural and religious interpretations which contrasted with the more dominant narratives of family unity (Norris 2009).

Their telling was undoubtedly facilitated by my being a woman, but more specifically a young, single, Western woman. My lack of husband, marked by the absence of a wedding ring which did not go unnoticed by the women, was drawn into the conversation with reference to how lucky I was not to have marriage complications coupled with advice to stay single. While an ‘other’ in terms of marital status, an apparent solidarity had been created though our shared gender. The experience that marriage created vulnerability was one these older women wanted to protect me from, irrespective of our different ethnic/cultural/religious backgrounds. While this aspect decreased my apparent ‘otherness’, it was emphasised by my position as a non-Muslim, non-Gayonese/Acehnese/Indonesian. For these women, being seen as a ‘good Muslim’ was essential to their social standing, reinforced by the ethnic complications that had been wrought by the civil conflict (Norris 2009). Yet as an ‘other’ I was an apparently safe ear to which their experiences could be told. Frequently, comments were made when others, including my co-researcher, had left the room, when the dictaphone was switched off or when physical props such as photographs or indeed coffee trees were available for the interviewee to facilitate the conversations. In doing so it was as if they were talking *to* rather than *with* someone, sharing their story with a neutral presence but only in a unilateral direction. But this potential assumption that I, like the photograph, was a neutral ear is untenable and not supported by my reflexive engagement.

I was brought up a Christian and I live in a country (UK) in which Islam is a politically and socially potent term, and where reference to women's position is subject to frequent social commentary (Poynting and Mason 2007). Yet I was born and brought up in a Muslim country and spent five years living in a predominantly Hindu one. Religious plurality, in short, is my background and with it an agnosticism tinged with deep interest in the nature of religious belief, including Islam. Concepts of the ‘good Muslim’ were of interest to me both academically and personally, but took on a different significance when combined with gender and my own social concepts of equality and marital relationships. While my gender and lack of husband (which was mirrored by my co-researcher) were particular assets in facilitating this topic, I am a woman from a very different cultural and social background. The combination of solidarity and ‘otherness’ resulted in a strong emotional engagement with the narratives. An extract from my diary written directly following one interview highlights my reaction:

Sat in the car fuming once again. This is so crap for women. How is it that a man can just leave his wife, does what he pleases with no social reprimand and she is left to manage the fields and do all the work in order to pay for his life with the new wife. This is grossly unfair. Once again I find myself slightly hateful towards this man – still thinking he was treating his wife a lot like a machine. I must take care of these thoughts, I am all over them, but it is hard to not want to punch him.

My engagement with this topic is clearly illustrated by my fuming body which wanted to vent its anger through a physical act of violence to the perceived perpetrator of wrong-doing. Also apparent are judgements based on my personal experiences and expectations of life and my own behaviour as a researcher. I express concern that my Western self may erupt, creating the potential that its distinctive ‘otherness’ would interrupt the connection that had been created through gender bonds. Consequently, I actively shielded part of myself from my participants, taking care to protect our relationship from views that were distinctly my own and an emotional response that was out of keeping with the current context (in terms of socially acceptable behaviour and my position as a researcher). This awareness of my ‘otherness’ resulted in feelings of vulnerability, but also shame. I was aware I was trying to shape myself to conform to acceptable local behaviour, while feeling dishonest for hiding myself and in doing so not demonstrating solidarity with these women as I would in my own familiar context.

The embodied experience of both my frustration and anger, which I consciously suppressed in public, and my sense of vulnerability highlighted something regarding the social experience of these marginalised voices (Sharma, Reimer-Kirkham and Cochrane 2009). The intersectionality between age, gender, martial position, religion, social discontinuity post-conflict, ethnicity and disability carved a uniquely vulnerable position for these women which I experienced viscerally. By exploring my anger and my gendered experience of living both within the UK as well as in this unusual research context, that vulnerability took shape. These women had not only been deserted by their husbands, but also by the society in which they lived. The need to remain within social boundaries prevented other women from aligning themselves with these women. My shame at inaction ignited awareness that I had also deserted them because of my concerns with emphasising my social ‘otherness’ and my need to collect data. This subsequently assisted my understanding that expressing dissatisfaction with their position would have potentially positioned these women as ‘others’ within their community and hence their need to confide where there was apparent space; myself, the trees, the photographs.

This example once again emphasises the fluctuating influences on embodied identity, but also how this impacted on the research itself. The participants' and my own contradictory utilisation and camouflaging of my ‘otherness’ facilitated the story. By taking note of my emotional response, my appreciation of their story altered. When removed from the constraints of the field, those emotions lingered and grew, directly influencing what I chose to report. These stories were a minority finding, but I chose to present the case of one of these women while others were omitted, writing ‘because of its salience to the individual affected it is important to report’ (Norris, Allotey and Barrett 2012: 832). While I am confident of the importance of these stories for the participant, a more honest presentation would also have acknowledged their importance to me, one which my embodied frustration forced me to consider, but also one which is born from my unique embodied presence and negotiations of status within the community.

## Embodiment

The final area of reflection focuses on the very site of embodiment, the human body. Seymour (2007: 1195) notes that ‘fieldwork is predominantly body work … bodies bearing messages and messages bearing bodies'. In the case of Seymour (2007), alongside other authors (Brown and Boardman 2011), the disabled body is the focus of enquiry. Yet the body, everybody's body, holds visible stories which can be interpreted by those who are in its presence, and whose physical presence is interpreted and lived by the persons themselves (Turner and Norwood 2013). My aim in the following section is to trace the outline of my actual physical being and my being within it, in a world which views bodies in a different way to my own perceptions and understandings. In doing so, the intersectionality between environment, roles, capability, history and social value are fore-fronted, with gender, age and ethnicity overshadowed. Once again it becomes apparent that my embodiment has a critical role to play in the narratives that evolve.

### Embodied presences: being the body

**Figure 1.**
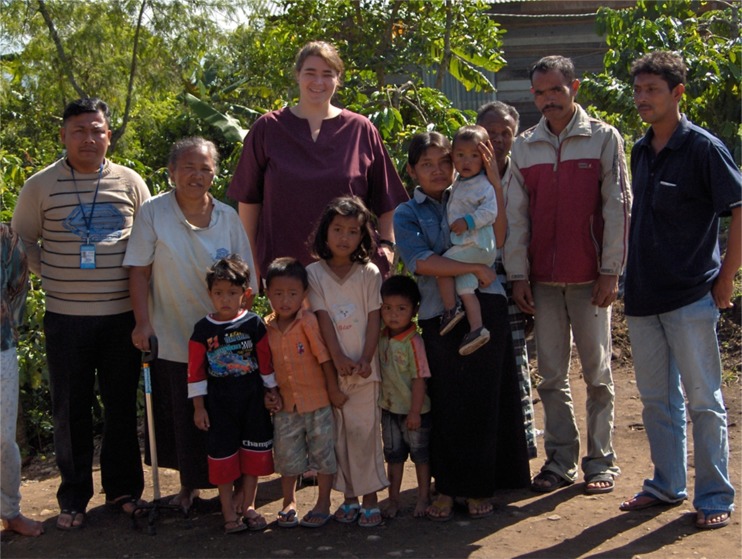
My physical presence (centre) in relation to one participant (left) with her grandchildren.

In the UK, at nearly six foot I am above average height and considered obese. I use what Warin and Gunson (2013) call the ‘O’ word intentionally to indicate my size, but also to draw attention to the social and moral associations that it entails within the dominant UK culture. I am a fit and healthy young(ish) woman who grapples daily with the knowledge that I am labelled, as Warin and Gunson (2013) describe, a moral failure, a failed citizen – social narratives I have absorbed. But my embodiment of failure is drawn from and lived in a particular time and place, where specific cultural values of the ideal body dominate. Indonesians, according to some scholars, share some of these views with the ideal body considered svelte, supple and lean (Ferzacca 2001), a view at odds with my robust and somewhat rotund body. Consequently, even before arriving in Aceh I was aware that my physical stature could influence the research process. I questioned whether my height and size would be intimidating and whether negative social judgement would follow me to the field. On arrival my physical ‘otherness’ was impossible to ignore, I was not only white but also compared to my participants I was huge (*see* Figure 1). I therefore considered my presence as an ‘unexpected body’, one which disrupts normalcy for the interviewees (Kannen 2013), and I questioned what impact that disordering of expectations would have on the narratives produced. Over the course of the fieldwork I was further alerted to the internal experience of my physical presence and its impact on myself and the interactions with participants.

Initially it became apparent that the description of the ideal body, drawn from fieldwork in Java, was not completely shared with participants in this study. For the vast majority of people, life in this mountainous region is physically demanding. Small holdings (*kebun*) were owned by most families and so farming was an everyday occupation for both men and women. I heard and observed these physical demands but also experienced them while walking through the coffee plantations, to people's homes and assisting with the clearing of scrubland when neighbours required the assistance. Within that context I was an interesting physical proposition. I was considered ripe for working in the *kebun*, which frequently came up in conversation, but my physical presence and embodied experience of the environment were also potentially active players in the discussions regarding participants' lives.

Stroke typically results in physical as well as many other deficits, as may ageing more generally. It was therefore unsurprising that concerns with the physical were articulated both through the interviews and in the photographs taken by the stroke survivors. Of interest, however, was the dominance of the physical (over and above the social, for example) and its nature as these concerns were predominantly functional rather than those with either cosmetic implications or how the sense of self had been altered as a consequence of stroke (both frequently mentioned in other stroke literature, *e.g.* Ellis-Hill, Payne and Ward 2000; Wiles *et al.* 2004). The participants discussed in depth and illustrated the activities that they were required to do, which frequently involved those activities that they had related to my perceived capacity. They also considered the reliability (or lack of) of their bodies which now prevented them from independently participating in desired physical roles and in some cases the real physical roles required in order to sustain their position as an active member of their family (*see* Norris, Allotey and Barrett 2012). In this region of Indonesia, retirement was not a held concept and physical work regardless of age was a required part of everyday life. The only participants who diverged from this position were two who remained ill following their stroke and subsequently focused more on end-of-life issues relating to religious obedience and duty to their spouse.

These conversations were often held in relation to my physical stature and capacity, thereby explicitly drawing me and my body into the story of their stroke. Yet in the telling and performing the participants' story my body became a prop both literally and metaphorically. I was a physical reminder of what they wished to be and had lost due to their stroke, but I was also physically utilised within the telling process. My legs and back were slapped to illustrate their strength, and my forearms pinched. Jokes were made about how I would be able to lift them with one hand, invitations made to come and work in the field with them. This performative aspect of interviews has been discussed in depth elsewhere (Hyden and Brockmeier 2008), but my focus here is less on the performance of the interviewees and more on my improvised delivery, in which my bodily presence, inscribed by my own perceptibly unique cultural and social history, was launched into the heart of a story for which I was somewhat unprepared. Of note in these interactions was the lack of reference to my age, gender or ethnicity, which were not raised within this context by participants. It would appear that my literal physical presence and its association with their current physical experience of life post-stroke and their wishes for the future resulted in these apparent indications of ‘otherness’ to be somewhat hidden, irrelevant or even meaningless.

Being seen as physically robust with a useful body was a counter-narrative to my everyday life within the UK. The moral baggage of obesity appeared to hold no sway here. These interactions did not, however, normalise me, but de-normalised me in a different and positive context. So the ‘unexpected body’ was not just mine for the interviewees, but how mine was now constructed by me. This changing relationship with my own body was, I believe, directly and indirectly an active feature in the research process. I explicitly, although not necessarily consciously, encouraged the physical performances and through that a focus on physical functionality. I allowed my body to be handled and did not stifle conversations regarding my regular swimming, walking and martial art training which were observed and subsequently shared in the community. I was becoming more comfortable with this new self-image, and as it appeared to facilitate a specific rapport with the participants I utilised it as a research tool. More indirectly, my body was simply present. I moved confidently and fluently through the terrain surrounding the participants' home, sitting on the floor and rising with ease, all activities my participants had once taken for granted but could no longer. Such physical differences are not uncommon in gerontological research.

Yet there was also an opportunity for me to refocus on my ‘normative’ Western self. One conversation around caring resulted in three adult daughters discussing their difficulty with toileting their mother, whom they needed to carry. I was sitting on the floor next to the mother and I looked at her to consider the impact of this difficulty. The eldest daughter immediately followed this with a comment that the daughters should be grateful their mother was not my size as they would never manage. This led to much laughter and agreement from the whole room including myself. Despite the jovial turn, my feeling of physical vulnerability and negative social judgement was immediate and internally I slumped, the awareness of my own weight bearing heavily. But within the context of the juxtaposed position of positivity, this awareness was incredibly valuable. My instant change in confidence and self-worth that resulted assisted me in considering not just the functional difficulties that the interviewees with stroke articulated and the practical implications for their family, but also the potential emotional consequence of unreliability, of social judgement, of the participants' embodying a distrust of their body in everyday life and the literal physical weight of requiring care. These insights encouraged an exploration of these topics in subsequent interactions and hence a development in the data collected. They also facilitated a deeper consideration of the concept of unreliability in the analysis which resulted in a more nuanced presentation of the consequences of stroke and one that moved beyond the initial functional description (*see* Norris, Allotey and Barrett 2012).

Sharma, Reimer-Kirkham and Cochrane (2009) suggest that the researcher's experience of an environment can assist in understanding the participant's world by compelling the researcher to consider how the participant's body interacts in the participant's world. My experience emphasises the potential insights gained from being in the environment physically, but also how that physical being is constructed and experienced socially and morally, by oneself, by the participants and within the shared encounter that results. Furthermore, experiencing a shift in my own body awareness created further fields of possible enquiry, enhancing an appreciation of the complexity of the participants' construction of their body.

## Discussion

While the examples described previously are drawn from a specific place and subject, the methodological issues they raise have significance beyond Indonesia and stroke and are pertinent to gerontological research more broadly and specifically when ethnicity is deemed a feature. All three examples in isolation, but particularly when considered together, illustrate that an individual's embodied, performed and social identities, including those of the researcher, are multiple, specific in time and place, and evolve and fluctuate through interaction. I am not just a white person, nor a researcher, nor a student, nor a woman, nor British, nor a daughter, nor bereaved, nor obese, nor youthful, able and strong, but an embodied combination of all of those things plus several more created and formed and reformed through my lifecourse and through the evolution of an interview and its analysis. The examples also illustrate the meshing of researcher and participant intersubjectivities across apparent ethnic and age boundaries and how these combined embodiments have a direct and indirect influence on the production, recording and representation of data. It is these last points which highlight the critical need for gerontologists to engage more deeply with reflexive embodiment (Turner and Norwood 2013). It is also important to note that by engaging with my embodiment, how the participants also utilised, reacted to and involved me in their storytelling becomes more explicit. Participants are not passive in the encounter between themselves and the researcher and a focus on intersubjectivity must acknowledge the mutual interdependence of story creation (Tarrant 2013). By encouraging a focus on reflexive embodiment, I am not giving primacy to the researcher alone but highlighting the need to acknowledge the researcher's moment to moment as well as the broader presence in the illumination of the participant's world (Doyle 2013; Finlay 2005).

In this last section, I focus on three methodological concerns, drawn from this research, which have specific relevance to the endeavour of exploring ageing and ethnicity.

### Environment

The significance of my personal interaction with the physical environment, including the participants, was highlighted as an important influence on the research. However, such insights into the researcher's physical capabilities are rarely presented in gerontological research despite the fact that the participants' physical interaction with their environment is often a topic of detailed conversation. Subsequently, we cannot consider how a researcher's physical presence and performance potentially influenced or not the participants' narratives on the vulnerabilities wrought by their failing bodies (Clarke and Bennett 2013; Clarke and Griffin 2008), for example, or in contrast the sustenance of their vibrant physicality (Phoenix and Smith 2011).

While considering the researcher's interaction with the environment should be of general interest within gerontology, research indicates that this may have specific pertinence in ageing and ethnicity. Zubair, Martin and Victor (2012*a*, 2012*b*), for example, demonstrate how use of physical space and the researcher's presentation within that had a critical impact on their interaction with the participants, including access.

### Emotions

Gerontologists on the whole research an area yet to be experienced by them, but which lies in their potential future. Thus, the stories heard from participants may in time be their stories. Consequently, the researcher has a personal connection with hopes and fears which are alive in the research. Stories of abuse (*e.g.* Beaulaurier *et al.* 2005; Hightower, Smith and Hightower 2006), ageism (Minichiello, Browne and Kendig 2000), decline (Clarke and Bennett 2013), loneliness (Stanley *et al.* 2010), racism (Worth *et al.* 2009), as well as those of ‘successful ageing’, are not heard in a vacuum and are likely to impact on those who share them. As my reflections indicate, that impact has the potential to influence what we present. Yet in the research cited above, this apparent emotional vacuum is left unexposed. It is my contention that a consideration of emotions within the embodied researcher affords the opportunity to enhance the honesty of the stories we present by making our presence more explicit.

### Space

The final area is that of the potentialities of space within the researcher–participant encounter. A focus on difference as a predominantly negative trait has resulted in the exploration of space within difference to be neglected (Ben-Ari and Enoush 2013). In previous research on ageing and ethnicity, awareness of that space has resulted in adaptations and accommodation to facilitate trust (Hall 2004; Zubair, Martin and Victor 2012*a*, 2012*b*). Other examples, alongside those presented in this paper, illustrate how apparent spaces, including those created by differences in ethnicity or age, dissolve when other more pertinent social connections can be brokered (Tarrant 2013; Wray and Batholomew 2010). A focus on specific features, such as occurs in age and ethnic matching, risks ignoring other aspects of the researcher's identity as well as good social rapport and understanding (even if not social similarity) being undeveloped (Carter and Bolden 2012; Sharma, Reimer-Kirkham and Cochrane 2009).

Unfortunately, space is frequently silent in gerontological literature and the ethnicity of the researcher managed through apparently simple statements such as the ‘Arabic fieldworker’ (Shanley *et al*. 2013). Likewise, Romo *et al.* (2013) discuss the linguistic capacity and ethnic background of interviewers and steps to ensure linguistic equivalence in their study on healthy ageing for disabled ethnically diverse elders. However, no other descriptors or potential influence are noted or any other subjectivities which the interviewers undoubtedly embodied, leaving the reader ignorant of how the shared ethnicity impacted on the study.

Researcher and participant difference is inevitable and political and social histories such as colonisation, migration and residence status, related power and potential language differences, create issues that cannot be ignored. However, these potential spaces must not be subsumed in crude categories but methodologically explored and exposed both to the challenges and possibilities they create. As gerontology moves forward in the field of ageing and ethnicity, a focus on space would serve to facilitate this exploration.

Accounting for environment, emotions and intersubjective space, as illustrated in this paper, can be enhanced by engaging with the embodied researcher. Sharma, Reimer-Kirkham and Cochrane (2009: 1643) note that ‘bringing the body back in means taking risks to be accountable to how one's bodily self can impact qualitative inquiry’. In line with Clarke (2012), I invite gerontologists to take that risk and for those involved in ethnicity and gerontology combined to grasp the opportunity afforded to them to explore apparent spaces to see what lies within.

## Conclusion

Through a reflexive embodied account of cross-generational and cross-ethnic research, this paper has sought to challenge simple and static constructions of ‘self’ and ‘other’ and a differential positioning of researchers and participants within the processes of research and knowledge production. Examples from the field have served to illustrate that a simple focus on divergent constructions of research roles and personal/social identities belies the complex interactions that occur within interviews in which multiple identities are performed, negotiated and reconstructed. The paper has further illustrated the role of embodiment in the construction of particular narratives, the production of knowledge and specific research relationships. Through this, the co-positioning of the researchers and the participants as both active co-participants within the research process and co-producers of knowledge is highlighted. The relevance of engaging with reflexive embodiment is thus emphasised as an important methodological concern generally, but specifically within the cross-generational ethnic context.
